# Translation and adaptation of the stroke-specific quality of life scale into Swahili

**DOI:** 10.4102/sajp.v79i1.1847

**Published:** 2023-03-29

**Authors:** Emily M. Nyanumba, Joseph M. Matheri, Nassib Tawa, Patrick M. Mburugu

**Affiliations:** 1 Department of Physiotherapy, Faculty of Rehabilitation, Kenya Medical Training College, Nairobi, Kenya; 2 Department of Physiotherapy, Faculty of Rehabilitative Sciences, Jomo Kenyatta University of Agriculture and Technology, Nairobi, Kenya; 3 Centre for Research in Spinal Health and Rehabilitation Medicine, Department of Rehabilitation Sciences, College of Health Sciences, Jomo Kenyatta University of Agriculture and Technology, Nairobi, Kenya; 4 Department of Child Health and Paediatrics, School of Medicine, Jomo Kenyatta University of Agriculture and Technology, Nairobi, Kenya

**Keywords:** stroke, SSQOL scale, translation, cultural adaptation, pre-validation, Swahili

## Abstract

**Background:**

Stroke care requires a patient-centred, evidence-based and culturally appropriate approach for better patient clinical outcomes. Quality of life necessitates precise measuring using health-related quality measures that are self-reported and language appropriate. However, most of the self-reported measures were devised in Europe and therefore not considered contextually appropriate in other settings, more so in Africa.

**Objectives:**

Our study aimed to produce a Swahili version by translating and adapting the stroke-specific quality of life (SSQOL) scale among people with stroke in Kenya.

**Method:**

We used a questionnaire translation and cross-cultural adaptation. The pre-validation sample of 36 adult participants was drawn from 40 registered people with stroke, from the Stroke Association of Kenya (SAoK). Quantitative data were collected using both English and Swahili versions of the SSQOL scale. The mean, standard deviation (s.d.) and overall scores were calculated and are presented in tables.

**Results:**

The back translation revealed a few inconsistencies. Minor semantic and equivalence alterations were done in the vision, mood, self-care, upper extremity function and mobility domains by the expert review committee. Respondents indicated that all questions were well-understood and captured. The stroke onset mean age was 53.69 years and the standard deviation was 14.05.

**Conclusion:**

The translated version of the Swahili SSQOL questionnaire is comprehensible and well-adapted to the Swahili-speaking population.

**Clinical implication:**

The SSQOL has the potential to be a useful outcome measure for use in Swahili-speaking patients with stroke.

## Introduction

The deteriorating health-related quality of life (HRQOL) burden in the stroke population is increasing globally, raising public health concerns (Muralidharan et al. 2019; Shakya et al. 2019). Medical advances in the management of patients with stroke and demographic changes witnessed mainly in populations of lower-middle income countries have led to a decrease in stroke deaths (6.5 million), an increase in stroke survivors (25.7 million) and disability-adjusted life years (DALYs) (113 million) (Donkor 2018).

Stroke has been and remains a major cause of adult activity limitation and participation restriction, which poses a challenge to the HRQOL among people with stroke (Donkor 2018; Gorelick 2019; Odetunde, Akinpelu & Odole 2017). Stroke prevalence is estimated at 0.5% globally with lower-middle-income countries recording high numbers of stroke deaths and DALYs (4.85m and 91.4m) as compared to high-income countries (1.6m and 21.5m) (Donkor 2018; Odetunde, Akinpelu & Odole 2018; Shakya et al. 2019; Tawa et al. 2021). The global detrimental effects of stroke on activity and participation are the reason why people with stroke seek rehabilitation services to improve symptoms, function and enhance their well-being (Gbiri & Akinpelu 2013; Muralidharan et al. 2019; Winstein et al. 2016).

The rising global burden of poor HRQOL among people with stroke has seen the emergence of efforts to integrate precise and valid patient-centred outcome measures to enhance rehabilitation and as such improve stroke outcomes. Perhaps that is the reason why people with stroke seek not only good medical and surgical care but also quality rehabilitation services (Donkor 2018; Moshki et al. 2019; Puthenpurakal & Crussell 2017). The health-related patient-reported measures are subjective as they quantify outcomes of an individual’s everyday life in their setting to inform rehabilitation plans (Gbiri & Akinpelu 2013; Kyte et al. 2015).

However, despite the increased global implementation of self-reported standardised measures in rehabilitation (Bowden & Fox-Rushby 2003; Kyte et al. 2015; Ganvir, Harishch & Kunde 2018; Hawkins, Elsworth & Osborne 2018), there is an underutilisation of patient-reported outcome measures (PROMs) in Kenya. This has been associated with limited knowledge of the importance of PROMs and also the limited existence of contextually appropriate measures. Again, the large number of PROMs that exist has been developed and tested in high-income countries such as the United Kingdom, Australia and America (Hall et al. 2018). Notably, the culture and language in these countries are different from that of Africa in relation to disease expression (Beaton et al. 2002; Guillemin, Bombardier & Beaton 1993). This, therefore, restricts the utilisation of PROMS in multi-ethnic and multicultural contexts (Odetunde et al. 2018), like that of Kenya. Global sub-cultural populations are typically defined in terms of lifestyle and dialect and hence the complexity of direct administration of PROMs developed elsewhere in other contexts (Beaton et al. 2002; Tawa et al. 2021). Either way, modern healthcare practice is appealing for a global response to cultural adaptation of PROMs to enhance the quality of care post-stroke (Hawkins et al. 2020).

Cultural adaptation as a process ensures a clear understanding of self-reported clinical questionnaires by the consumers (Beaton et al. 2002; Hall et al. 2018). The adaptation is not limited to translation to another language but also considers the contextual, semantic, item and conceptual equivalence. The contextual equivalence ensures the words used in the translated version echo the targeted culture, whereas semantics maintains the meaning of words in the translated version as in the original version. Item equivalence ensures translated documents measure the same construct as the source language and conceptual equivalence warrant good content capturing (Hall et al. 2018; Hawkins et al. 2020). The PROM that is the focus of our study is the original English stroke-specific quality of life (SSQOL) scale that was devised over two decades ago by William et al. (1999). The SSQOL scale precisely measures the quality of life of people who have been diagnosed with a stroke and possibly the reason for its comprehensive utilisation as evidenced in its diverse translations into more than 20 languages (Muralidharan et al. 2019; Odetunde et al. 2017).

However, there is a paucity of translation and cultural adaptation of the SSQOL scale into Swahili in sub-Saharan Africa and more so in Kenya despite Swahili being the most widely spoken language in sub-Saharan Africa (Kibui 2014; Michira & Iribemwangi 2014). Even so, researchers (Donkor 2018; Odetunde et al. 2018; Shakya et al. 2019) have revealed that lower-middle-income countries are the most affected by stroke, thus the need for utilisation of HRQOL measures that are language-appropriate for better outcomes. Therefore, translation and adaptation of the SSQOL scale into Swahili is fundamental in the Kenyan context as well as the entire sub-Saharan Africa to fill the gap. This was in line with scenario ‘E’ of the cross-cultural adaptation scenarios described by Beaton et al. (2002). Scenario ‘E’ states that a questionnaire can be adapted to be used in another country and in another language as in the case of our study. Thus, this is the reason for our study that has translated and adapted the SSQOL scale into Swahili.

Our study’s specific objectives were:

- To translate the original English of the SSQOL scale into Swahili
- To culturally adapt the translated Swahili version of the SSQOL scale into a Kenyan clinical context.

## Methods

A questionnaire translation and cross-cultural adaptation design was utilised. The SSQOL scale was translated and adapted into Swahili among people with stroke in the Nairobi metropolis, Kenya. Like in other studies (Odetunde et al. 2018, 2020; Wayessa et al. 2022), the translation and adaptation process was conducted in five steps under the American Association of Orthopaedic Surgeons (AAOS) published guidelines that comprised: forward translation, synthesis, backward translation, expert review committee and pre-validation (Beaton et al. 2000, 2002). Of the five steps recommended by Beaton et al. (2002), steps I–III entailed the translation process, step IV cultural adaptation and step V, pre-validation.

The SSQOL is a disease-specific patient-reported outcome measure designed to assess the quality of life among people with stroke (Williams et al. 1999). It consists of 49 items grouped into 12 domains of mobility, work, upper extremity function, thinking, personality, mood, family roles, social roles, energy, self-care, vision and language (Odetunde et al. 2018; Williams et al. 1999). Items per domain range between 3 and 6, and their responses are in the form of a Likert scale with the worst affected item being scored 1 and the not affected item scoring 5. The maximum overall score of the questionnaire is 245, whereas the minimum is 49. A score of 60% of the maximum score denotes a low quality of life (Chandran et al. 2017).

### Translation process

Forward translation was the first step in translating the SSQOL scale English version into Swahili. Notably, the SSQOL scale is open to the public, therefore, guaranteeing automatic permission for translation and adaptation (Williams et al. 1999). In this step, two consenting independent bilingual Swahili linguists with good knowledge of English, as per the published guidelines of the AAOS (Beaton et al. 2002), translated the (12) domains of the 49 items, word by word, into Swahili. Translation and adaptation of a questionnaire being new knowledge in Kenya caused a limitation of sector-specific translators although the independent translation is a requirement of this process. One of the linguists was a high school Swahili teacher and the other a Swahili editor from a leading publisher (Kenya) (Odetunde et al. 2018). The Swahili teacher was aware of the purpose of the process to safeguard the clinical aspect of the wording while the Swahili editor was blinded such that he was not provided with information regarding the impression and the purpose of translating the SSQOL scale (Odetunde et al. 2018). The two linguists each generated a written report of the process and two translated documents of Swahili 1 (S-1) and Swahili 2 (S-2).

The two Swahili documents (S-1 and S-2) were then reconciled, as recommended in step two of the guidelines (Beaton et al. 2000, 2002; Ganvir et al. 2018) by the two linguists and a senior physiotherapist from Kenyatta national teaching, research and referral hospital. The senior physiotherapist was conversant with translation studies, and he, therefore, acted as a scribe to guarantee the semantic and conceptual equivalence of the reconciled document (Odetunde et al. 2020). All items and specific words that were not in line with the original words as used in the scale were acknowledged clearly and a resolution was sought through consensus between the two linguists and the senior physiotherapist (Table 1). This reconciliation step produced one translated Swahili version questionnaire called Swahili 1 and 2 (S-1 and S-2) as recommended by Beaton et al. (2002).

**TABLE 1:** Consensus Swahili translation.

Issue: (specify item # and describe issue)	Forward translation	Problem	Resolution
Family roles 3. ‘My physical condition interfered with my personal life’	T-S1: *Hali yangu ya kimwili ilivuruga maisha yangu ya kibinafsi*	The word condition was translated as ‘*kimwili*’ and ‘*kiafya*’ and interfered as ‘*ilivuruga*’ and ‘*iliathiri*’	The team settled on the commonly used words. ‘*kiafya*’ and ‘*iliathiri*’
	T-S2: *Hali yangu ya kiafya iliathiri maisha yangu ya kibinafsi*		
‘Mobility’	T-S1: *Uwezo wa kutembea*	The word ‘mobility’ was translated as ‘walking’ which did not mean mobility	It was translated well to ‘moving the body’
	T-S2: *Kutembea*		
‘Thinking’	T-S1: *Uwazaji*	The first translation meant ‘thoughts’ and the second ‘thinking’	Translation 2 was adopted as the word ‘*fikra*’ was commonly used
	T-S2: *Fikra*		
‘Upper extremity function’	T-S1: *Kazi ya Upeo wa juu*	It was difficult to translate the word ‘extremity’ as there is no *kiswahili* word for ‘extremity’	It was translated well to ‘hand function’ in order to maintain meaning
	T-S2: *Matumizi ya Sehemu ya*		
	*juu ya mwili kama vile mikono*		
5. ‘Did you have trouble opening a jar?’	T-S1: *Jagi*	There was no *kiswahili* word that could fit the word ‘jar’	The word ‘jar’ was translated well to ‘jug’
	T-S2: *Birika*		

*Source*: Adapted from Odetunde, M.O., Odole, A.C., Odunaiya, N.A., Odetunde, N.A., Okoye, E.C., Mbada, C.E. et al., 2020, ‘Cross-cultural adaptation and validation of the IGBO language version of the stroke-specific quality of life scale 2.0’, *Pan African Medical Journal* 37(111), 1–14. https://doi.org/10.11604/pamj.2020.37.111.19557

T-S1, translation Swahili 1; T-S2, translation Swahili 2.

The reconciled S-1 and S-2 document was again back translated to English in the third step to ensure the message in the two versions; that is, the English and the translated Swahili (S-1&2) versions of the SSQOL scale were maintained and corresponding (Odetunde et al. 2018). As in the previous step, the back translation was also accomplished by two different consenting English linguists from a leading publisher (Kenya) although both were naïve to the forward translation process.

The two linguists generated their own written reports of the process and provided one document each, of back translation Swahili one (BTS-1) and back translation Swahili two (BTS-2), respectively, as recommended by Beaton et al. (2002). Further, the two linguists were joined by the first author (acting as a linguist) and together they analysed the inconsistencies in the two documents and by consensus resolved them appropriately producing one version of the back translation of Swahili 1 and 2 (BTS-1 and BTS-2) (Hawkins et al. 2020).

### Cultural adaptation process

The three documents, that is, the Swahili version (S-1 and S-2), the back translated version (BTS-1 and BTS-2) and the original English version of the SSQOL questionnaires, were subjected to step IV, for the cultural adaptation process referred to by a committee of experts (Hall et al. 2018). The committee comprised of two linguists (one from the forward and the other from the back translation), two neuro-physiotherapist lecturers (Jomo Kenyatta University of Agriculture and Technology [JKUAT] and Amref International University (AMIU) universities), a senior physiotherapist (different from the one who reconciled S-1 and S-2) from Memorial Forces hospital Nairobi with over 17 years of stroke rehabilitation experience and the first author acting as a secretary and a scribe as recommended by Beaton et al. (2002). The experts also consented prior to the process of cultural adaptation.

The expert review committee examined the 49 items of the questionnaire for contextual appropriateness, transparency, relevance and equivalence to the target population and ensured that the phrases and words were as used in everyday language (Hall et al. 2018; Hawkins et al. 2020). Sensitive words such as sexual intimacy were treated carefully to ensure justice and no offence or violation when being applied as recommended by Ganvir et al. (2018). The first author documented all the items, phrases and words not corresponding with the original questionnaire and the process undertaken to come to a consensus to produce the pre-final Swahili version (PFSV) as recommended by Odetunde et al. (2018).

### Pre-validation of the pre-final Swahili version

Finally, the PFSV and the original English version of the SSQOL questionnaires were administered to eligible people with stroke for pre-validation purposes (Pedersen et al. 2018). This was the ultimate stage of the translation and adaptation process that focused on guaranteeing the content and face validity of the PFSV questionnaire as recommended by Beaton et al. (2002).

Data were collected from the Stroke Association of Kenya (SAoK) situated within the Nairobi Metropolis. The SAoK is a registered organisation with an aim of rebuilding people with stroke by offering outpatient rehabilitation and wellness services to people with stroke. The association has more than 100 members with stroke, but only about 40 of them are appropriately registered with the association.

Therefore, all 40 registered members with stroke formed the study population.

The formula, (*n* = *N*/1+*N* (*e*)²) by Yamane (1967) was considered in calculating the pre-validation sample as shown below:


$$\begin{array}{l} n=40/1+40(0.05{)}^{2}\\ \;=40/1.1\\ n=36 \end{array}$$ [Eqn 1]

The calculated sample size was therefore 36 adults with stroke. The sample size was within Beaton et al.’s (2002) recommendations of between 30 and 40 participants in pre-validation studies of this nature, to enable a comparison of the responses of the two versions of the SSQOL questionnaires. A convenience sampling technique was used to recruit consenting people with stroke during 1 month starting from 16 March 2022 to 14 April 2022.

### Data collection

Data were collected by the first author and a research assistant using both the SSQOL English (E) and SSQOL Swahili (S) versions of the questionnaires. The Swahili version was assigned odd numbers that is number 1, 3, 5 and so forth while the English version was assigned even numbers that is number 2, 4, 6 in that order, respectively. All the patients at the waiting bay were briefed orally concerning our study and informed that it was voluntary.

Participants were also clearly informed that all the information given would be confidential (Odetunde et al. 2020). Thereafter, the files of those who volunteered to participate were checked to ascertain eligibility. Participants in the acute and severe stages of stroke, and those with a stroke diagnosis of 1 month or less as well as those with difficulties in reading, writing and understanding English or Swahili were not included.

Like in most studies (Odetunde et al. 2018, 2020), eligible participants diagnosed with either ischaemic or haemorrhagic stroke were given a written explanation about our study and signed written consent prior to completing the questionnaire. Consenting adult participants were then recruited systematically after receiving physiotherapy services. Each participant was issued with one questionnaire such that the first participant was issued with questionnaire number 1, which was SSQOL Swahili version and the second participant received questionnaire number 2, which was SSQOL English version. Then the third participant was issued with questionnaire number 3, which was SSQOL Swahili version and so forth, until the last questionnaire was issued as recommended by Odetunde et al. (2020). Participants completed the questionnaires in a private room that was set aside for the exercise. This was achieved by giving participants temporary research numbers for identification purpose.

Further, each respondent was taken through a face-to-face cognitive interview immediately after completing the questionnaire. The purpose of the interview was to assess the relevance, clarity and ambiguity of the items, as well as any important questions that may have been omitted and needed to be included as recommended by Bowden and Fox-Rushby (2003) and Goerman and Caspar (2010). All concerns raised were investigated and captured in the second meeting of experts producing a final Swahili version (FSV) as recommended by Beaton et al. (2002).

### Variables

The independent variable was the original English SSQOL scale, whereas moderating variables were forward translation to Swahili language, backward translation of the Swahili translated version to original English, adaptation of the translated Swahili version and the pre-validation of the Swahili version. The dependent variables comprised of the English and Swahili translators, the committee of experts and people with stroke. Our study’s outcome was SSQOL scale Swahili version.

### Descriptive analysis

Data were entered into an Excel sheet, cleaned and coded. The domain properties were inspected manually and the mean, standard deviation (s.d.), percentages, domain scores and overall scores of both English and Swahili versions of the SSQOL questionnaires were analysed through the Statistical Package for Social Sciences (SPSS) version 26 and presented in tables. There were no missing data in all the domains.

### Ethical considerations

Approval to conduct our study was granted from the Jomo Kenyatta University of Agriculture and Technology (JKUAT) Institutional Ethics Review Committee (IREC) (reference no.: JKU/IERC/02316/0488) and National Commission for Science, Technology and Innovation (NACOSTI) (reference no.: NACOSTI/P/22/15664). Permission to collect data was granted by the manager of the Stroke Association of Kenya.

## Results

### Findings of the translation process

The reconciliation of Swahili 1 and 2 documents had a few words that were difficult to translate, and other words had different meanings but were translated as similar. The reconciling team re-investigated those words and phrases and by consensus resolved them as summarised in Table 1.

### Expert committee findings

The experts’ first committee meeting noted that a good number of the items in the Swahili translation of SSQOL questionnaire were relevant and well-captured as they appeared in the English SSQOL scale. This is also the case in other translations (Odetunde et al. 2018, 2020). On the other hand, back translation indicated some discrepancies of 15 words/phrases/statements to the original English SSQOL scale. The discrepancies ranged from omissions, replacement and contextual inappropriateness to the Kenyan culture. Therefore, they were corrected at the meeting, and a PFSV was produced as summarised in Table 2.

**TABLE 2:** Expert committee meetings findings on the Swahili version of stroke-specific quality of life scale.

Issue: (specify item # and describe issue)	Consensus translation	Back translation	Problem	Resolution
**Original version** The title: ‘stroke-specific quality of life scale’	*Hojaji ya viwango maalum vya ubora katika maisha*	Specific-quality of life questionnaire	The word ‘stroke’ was missing in the back translation	It was translated well to include the word stroke
**The scoring key** ‘Strongly agree’	*Ninakubali*	BTS-1/BTS-2: I strongly agree	The word ‘I’ was captured both in forward and back translation contrary to the original version	The experts agreed to retain the word ‘I’ as it captured the desired concept
**Energy** **2.** ‘I had to stop and rest during the day’	*Nililazimika kuacha shuguli zangu ili kupumzika*	BTS-1/BTS-2: I had to stop doing my work and rest during the day	The word my ‘work’ was included in forward translation which meant ‘occupation’ altering the meaning.	The expert committee accepted the addition but adopted the word ‘activities’ instead of ‘work’ so as to maintain meaning
**Family roles** 1. ‘I didn’t join in activities just for fun with my family’	*Sikujiunga na familia yangu kujiburudisha*	BTS-1/BTS-2: I didn’t join my family in leisure activities	The word ‘just’ was omitted in both translations and the word ‘fun’ was translated as leisure in both forward and back translation	The expert committee was in agreement
**Language** ‘Did you have trouble speaking clearly enough to use the telephone?’	*Je, ulikuwa na matatizo kuzungumza na simu*?	Did you have trouble speaking using a mobile phone?	Both forward and back translation captured ‘mobile phone’ instead of ‘telephone’ and also the words ‘clearly enough’ were omitted	The expert committee was in agreement as it captured the Kenyan context and was easy to understand.
5. ‘Did you have to repeat yourself so others could understand you?’	*Je, ulilazimika kujirudia ili watu wakuelewe*?	Did you have to repeat yourself so that people would understand you?	Both forward and back translation included the word ‘that’ and used the word ‘people’ instead of ‘others’	It was acceptable to the expert committee
‘**Mobility’**	*Kusongesha mwili*	Moving the body	The back translation captured the words ‘moving the body’ instead of ‘mobility’	The words moving the body were borrowed as there was no Swahili word for mobility. It was acceptable to the experts committee
1. ‘Do you have trouble climbing stairs?’	*Je, ulikuwa na matatizo ya kupanda ngazi*?	Did you have trouble climbing ladders?	‘Stairs’ was translated as ‘ladders’ which meant a different thing	The word ‘house’ was added before stairs so as to mean ‘house stairs’ and was acceptable
4. ‘Did you have to stop and rest more than you would like when walking or using a wheelchair?’	*Je, ulilazimika kupumuzika ukitembea au ukitumia kiti cha magurudumu*?	Did you have to rest while walking or using a wheelchair?	The words ‘more than you would like, stop’ were not captured in the forward translation	It was acceptable as the phrase still maintained meaning
**Mood** 5. ‘I was not interested in food’	*Sikuwa na hamu ya chakula*	I did not have appetite	Forward and back translation capture the word ‘appetite’ and not ‘interested’	It was acceptable by expert committee
**Self-care** **2.** ‘Did you need help eating? For example, cutting food or preparing food?’	*Je, ulihitaji usaidizi kula? Kwa mfano, kuchukua chakula kutoka kwa sahani au kuweka chakula kwa mdomo*?	Did you need help eating? For example, taking food from the plate or putting food in the mouth?	‘Cutting food’ was replaced with ‘taking food from the plate’ and ‘preparing food’ replaced with ‘putting food in the mouth’	It was acceptable as it was contextually and experiential appropriate
**‘Upper Extremity Function’**	*Matumizi ya mikono*	Hand function	Both translations used ‘hand function’ instead of ‘upper extremity’	The expert committee was in agreement as the word extremity did not have a Swahili name
6. ‘Did you have trouble opening a jar?’	*Je, ulikuwa na matatizo kufungua jagi*	Did you have trouble opening a jug?	Opening a ‘jug’ was not in line with hand function	The committee borrowed the word ‘bottle’ so as to maintain the meaning
**Vision** 1. ‘Did you have trouble seeing the television well enough to enjoy a show?’	*Je ulikuwa na matatizo kutazama televisheni vizuri*?	‘Did you have trouble watching television well?’	‘Seeing’ was both forward and back translated as ‘watching’, and some words like ‘well enough, to enjoy a show’ omitted	It was acceptable as ‘it was contextually appropriate
2. ‘Did you have trouble reaching things because of poor eyesight?’	*Je, ulikuwa na matatizo ya kufikia vitu kwakukosa kuona*?	‘Did you have difficulties reaching things for not seeing?’	Back translation not consistent with original version	The original English was ambiguous. This was translated again, for easy understanding and it was acceptable by the expert committee

*Source:* Adapted from Odetunde, M.O., Akinpelu, A.O. & Odole, A.C., 2018, ‘Cross-cultural adaptatiion and validation of the stroke specific quality of life 2.0 scale into hausa language’, *Journal of Patient-Reported Outcomes* 2(1), 1–14. https://doi.org/10.1186/s41687-018-0082-1; Odetunde, M.O., Odole, A.C., Odunaiya, N.A., Odetunde, N.A., Okoye, E.C., Mbada, C.E. et al., 2020, ‘Cross-cultural adaptation and validation of the IGBO language version of the stroke-specific quality of life scale 2.0’, *Pan African Medical Journal* 37(111), 1–14. https://doi.org/10.11604/pamj.2020.37.111.19557

BTS-1, back translation Swahili 1; BTS-2, back translation Swahili 2.

### Pre-validation and face-to-face cognitive debriefing findings

The PFSV was tested among the 32 adults with stroke who agreed to be in our study and comprised of 16 males and 16 females who were either good at English or Swahili. The face-to-face cognitive debriefing interview revealed that 46 items out of the total 49 were well-understood in the PFSV and that they were relevant and well-captured. Of the three that were not relevant was item number 1 of the self-care domain, which read ‘Did you need help in preparing food?’ translated as ‘*Je ulihitaji usaidizi kuandaa chakula*?’. The item was not applicable to two participants that is one male and one female. The reason given by the male participant is that, as a man he never prepared food as it was the role of his wife. The female participant as well, explained that since her childhood, she has never participated in household chores such as cooking.

Also, item number 4 in the mood domain that read, ‘I had little confidence in myself’ was not clear to most of the participants. Lastly, item number 2 of the vision domain that enquired ‘did you have trouble reaching things because of poor eyesight?’ was reported to be ambiguous by participants. As a result, the second expert committee meeting was confined to address the concerns. As such, the committee deliberated on the three items, and it was agreed that item number 1 of the self-care domain be maintained as it was to consider the young generation, which is the majority and most urbanised. For item number 4 of the mood domain, the words ‘in my life’ were added to read ‘*Nilijiamini kiasi tu kwa maisha yangu*’ back translated as ‘I had little confidence in my life’. Finally, in item number 2 of the vision domain, simple and few words were used to make it comprehensible. This exercise guaranteed the development of the FSV of the SSQOL questionnaire (Appendix 1).

The respondents’ gender was equivalent, stroke onset mean age in this study was 53.69 years, the s.d. was 14.05, a stroke duration of 4 months and an interquartile range of 1. The majority (84%) of the respondents were 40 years and above when diagnosed with stroke and a few (16%) were below 40 years. Most respondents (78.1%) had an ischaemic stroke while a few (21.9%) had a haemorrhagic stroke. Additionally, 37% of the respondents had weakness on the right side while 63% had weakness on the left side. Only 28% of the respondents had suffered a stroke 16 months or less previously, and 72% had suffered a stroke more than 16 months previously. Respondents working and in business after the stroke were 28% while 72% were not employed or in business. Details are summarised in Table 3.

**TABLE 3:** Social demographic characteristics of pre-validation data (*N* = 32).

Variables	Frequencies	Percentages
**Gender**		
Male	16	50.0
Female	16	50.0
**Stroke type**		
Haemorrhagic	7	21.9
Ischaemic	25	78.1
**Age at onset of stroke**		
18–39	5	15.6
40–49	8	25.0
50–59	9	28.1
60–69	4	12.5
≥ 70	6	18.75
**Affected side**		
Left	20	63.0
Right	12	37.0
**Stroke duration**		
1–16 months	9	28.1
17–30 months	5	15.6
31–47 months	4	12.5
> 48 months	14	43.8
**Work status before stroke**		
Employed	27	84.0
Unemployed	5	16.0
**Work status after stroke**		
Employed	9	28.1
Unemployed	23	71.9

*Source*: Adapted from Odetunde, M.O., Akinpelu, A.O. & Odole, A.C., 2018, ‘Cross-cultural adaptatiion and validation of the stroke specific quality of life 2.0 scale into hausa language’, *Journal of Patient-Reported Outcomes* 2(1), 1–14. https://doi.org/10.1186/s41687-018-0082-1; Pedersen, S.G., Heiberg, G.A., Nielsen, J.F., Friborg, O., Stabel, H.H., Anke, A. et al., 2018, ‘Validity, reliability and Norwegian adaptation of the Stroke-Specific Quality of Life (SS-QOL) scale’, SAGE Open Medicine 6, 205031211775203. https://doi.org/10.1177/2050312117752031

Respondents’ mean scores on the vision domain was high but lower in the energy, mobility, self-care, social roles, upper extremity and work domains in both versions of SSQOL scale, respectively (Table 4)

**TABLE 4:** Mean scores of SSQOL(E) and SSQOL(S) versions (*N* = 32).

Domains	English/Swahili	Mean scores	Variation
Energy	English Swahili	1.3 1.2	0.1
Family roles	English Swahili	1.5 1.2	0.3
Language	English Swahili	2.0 1.6	0.4
Mobility	English Swahili	1.0 1.1	0.1
Mood	English Swahili	2.2 2.8	0.6
Personality	English Swahili	1.7 2.1	0.4
Self-care	English Swahili	1.1 1.1	0.0
Social roles	English Swahili	1.2 1.3	0.1
Thinking	English Swahili	2.9 2.2	0.7
Upper extremity function	English Swahili	1.2 1.3	0.1
Vision	English Swahili	3.6 2.5	1.1
Work/productivity	English Swahili	1.0 1.0	0.0

*Source*: Adapted from Odetunde, M.O., Akinpelu, A.O. & Odole, A.C., 2018, ‘Cross-cultural adaptatiion and validation of the stroke specific quality of life 2.0 scale into hausa language’, *Journal of Patient-Reported Outcomes* 2(1), 1–14. https://doi.org/10.1186/s41687-018-0082-1; Odetunde, M.O., Odole, A.C., Odunaiya, N.A., Odetunde, N.A., Okoye, E.C., Mbada, C.E. et al., 2020, ‘Cross-cultural adaptation and validation of the IGBO language version of the stroke-specific quality of life scale 2.0’, *Pan African Medical Journal* 37(111), 1–14. https://doi.org/10.11604/pamj.2020.37.111.19557

SSQOL(E), stroke-specific quality of life English; SSQOL(S), stroke-specific quality of life Swahili.

Respondents’ mean overall total score on the SSQOL(E) was 82, s.d. of 31.04 and SSQOL(S) was 81, s.d. of 20.24 (Table 5).

**TABLE 5:** Respondents scores on SSQOL(E) and SSQOL(S) (*N* = 32).

English respondents	Scores	Swahili respondents	Scores
2.16.3.22	56	1.16.3.22	73
4.16.3.22	111	3.17.3.22	70
6.21.3.22	93	5.18.3.22	105
8.21.3.22	60	7.19.3.22	90
10.28.3.22	54	9.19.3.22	81
12.6.4.22	60	11.21.3.22	77
14.4.4.22	63	13.22.3.22	130
16.6.4.22	100	15.22.3.22	103
18.4.4.22	66	17.18.3.22	76
20.6.4.22	68	19.18.3.22	73
22.6.4.22	147	23.18.3.22	68
24.7.4.22	141	25.26.3.22	101
26.7.4.22	65	27.8.4.22	71
30.9.4.22	76	29.8.4.22	64
34.14.4.22	70	31.14.4.22	90
-	-	33.14.4.22	52
-	-	35.14.4.22	52

**Total scores**	**1230**	**-**	**1376**
**Mean scores**	**82**	**-**	**81**
**Standard deviation**	**31.04**	**-**	**20.24**

*Source*: Adapted from Odetunde, M.O., Akinpelu, A.O. & Odole, A.C., 2017, ‘Validity and reliability of a Nigerian-Yoruba version of the stroke-specific quality of life scale 2.0’, Health and Quality of Life Outcomes 15(1), 1–12. https://doi.org/10.1186/s12955-017-0775-9

SSQOL(E), stroke-specific quality of life English; SSQOL(S), stroke-specific quality of life Swahili.

## Discussion

There have been numerous translations and adaptations of the SSQOL questionnaire adopting AAOS recommended guidelines across different countries and cultures (Beaton et al. 2000, 2002; Odetunde et al. 2018, 2020; Wayessa et al. 2022). Related studies have revealed that most of these cross-culturally adapted questionnaires have substantively achieved the goal of relevance and comparability across cultures as has also been demonstrated in the FSV of the SSQOL scale (Ganvir et al. 2018; Pedersen et al. 2018; Sallam et al. 2019; Odetunde et al. 2018, 2020; Wayessa et al. 2022).

Swahili is also known as Kiswahili and is the lingua franca of Eastern Africa where it originated (Lisanza 2021). Swahili is not only a national and official language in Kenya but also in another 12 sub-Saharan countries and two Middle East countries (Kibui 2014). In addition, Swahili is one of the official languages in the African Union (Lisanza 2021). Close to 100% of all the 42 ethnic communities of Kenya speak Swahili and most importantly, about 200 million people speak Swahili worldwide (Lisanza 2021; Michira & Iribemwangi 2014). Therefore, this explains the need for this translation and adaptation of the SSQOL scale into this commonly spoken Swahili language in Kenya, sub-Saharan Africa and globally.

Like in most studies, the reconciling of the two Swahili documents produced in the forward translation process revealed that some words such as ‘mobility’, ‘upper extremity function’ and ‘jar’ were difficult to translate and therefore had to be borrowed. For instance, mobility was written as ‘*kusongesha mwili*’ (moving the body), upper extremity function ‘*matumizi ya mikono*’ (hand function) and jar as ‘*chupa*’ (bottle). The finding is similar to that of SSQOL Igbo version where extremity was translated as ‘ability to use a hand’ and jar as ‘bottle cover’ (Odetunde et al. 2020).

The backward translation of the Swahili version had some inconsistencies with the English version such that some words or phrases were omitted and others replaced, which were similar to previous translations (Odetunde et al. 2018; Pedersen et al. 2018). For example, in the mobility domain, item number 4 that enquired ‘did you have to stop and rest more than you would like when walking or using a wheelchair?’ and translated as ‘*Je, ulilazimika kupumuzika ukitembea au ukitumia kiti cha magurudumu*?’ was back translated as ‘did you have to rest when walking or using a wheelchair?’ The words ‘stop and more than you would like’ as they appeared in the original English version were apparently lost as the phrase was clearly understood and conceptually appropriate. This was similar to Guillemin et al.’s (1993) translation recommendations of avoiding two verbs in one sentence and using short and simple sentences to enhance comprehensibility.

Also, in the language domain, item number 2 that read ‘Did you have trouble speaking clearly enough to use the telephone?’, the words ‘clearly enough’ were also omitted in both translations and ‘telephone’ replaced with mobile phone. This was like item number 5 of the mood domain, which read ‘I was not interested in food’ and back translated as ‘I didn’t have appetite’. This was an acceptable change as the experiential and cultural equivalent was maintained following the translation guidelines by Guillemin et al. (1993) and Hall et al. (2018) of finding substitute words that better fit the setting.

Besides the omissions, some items lacked consistency in the backward translation. For instance, item number 3 of the mobility domain, whereby the word ‘stairs’ translated as ‘*ngazi*’ was back translated as ‘ladder’, which was not only inconsistent with the original English but also altered the concept of interest. This was, therefore resolved after a debate by the expert committee members by adding the word house before stairs so as to be consistent and concept appropriate. This finding agreed with a translation study by Wagner et al. (1999) where the word ‘*Mlima*’ (hill) was adopted to mean stairs.

Further, some aspects of some phrases also had certain discrepancies and more importantly lacked contextual appropriateness. For instance, item number 2 of the self-care domain that read ‘Did you need help eating? For example, cutting food or preparing food?’ forward translated as ‘*Je, ulihitaji usaidizi kula*? *Kwa mfano, kuchukua chakula kutoka kwa sahani au kuweka chakula kwenye mdomo*?’ and back translated as ‘Did you need help eating? For example, taking food from the plate and putting food in the mouth?’ The back translation aspect reading ‘taking food from the plate or putting food in the mouth’ was not in line with the source language. Similarly, the part reading ‘preparing food?’ was not in line with eating as preparing food was not a component of eating therefore rendering the phrase irrelevant, and even so, preparing food was already captured in item number 1 of the same domain and thus treated as a repetition. Again, the facet of ‘cutting food’ was not appropriate to the Kenyan context as it is not an everyday practice in Kenya to cut food with a knife; instead, people use their hands or spoons to take food from the plate. As such, the phrase was revised to ‘taking food from the plate or putting food in the mouth’. This was similar to previous translations (Odetunde et al. 2018, 2020) and also in line with Guillemin et al.’s (1993) recommendations of altering phrases to fit the concept and context of application.

In addition, the pre-testing findings revealed that three items were irrelevant, ambiguous and lacked clarity. For instance, item number 1 of the self-care domain that enquired about having trouble preparing food was irrelevant to one male and one female participant. The male participant purported that cooking was the role of his wife while the female participant reported that she had since childhood never participated in performing household chores. The finding partially agreed with that of Odetunde et al. (2018, 2020) where some men in Hausa and Igbo also argued that food preparation was women’s work. However, the committee of experts was reluctant to modify this item because this alteration may restrict its application to the young generation who are the majority and most urbanised. These findings concurred with those of a recent study by Wayessa et al. (2022). Item number 2 of the vision domain was also found to be ambiguous in that most respondents did not clearly understand the meaning. This finding agreed with those by Pedersen et al. (2018) that reported the ambiguity of the same item.

Thirty-two respondents completed both the original English version of the SSQOL and the PFSV of SSQOL questionnaires whereby 15 of the participants completed the English version and 17 the PFSV as shown in Table 5. This sample size was in line with Beaton et al.’s (2002) recommendations to include between 30 and 40 participants in studies of this nature in order to enable correlation of the responses of the two versions. The stroke onset mean age was found to be 53.69 years, s.d. = 14.05, with a median time of 4 months of stroke duration and an interquartile range of 1. These findings are similar to those of other studies that revealed people between the ages of 40 and 60 years are more affected by stroke (Ganvir et al. 2018; Pedersen et al. 2018; Odetunde et al. 2018, 2020). The majority (84%) of the respondents ranged between 40 and 69 years further confirming the aforesaid reports. Though the rate of stroke in young adults is increasing drastically, it is still recognised that the risk of stroke increases with age (Donkor 2018; Yangatimbi et al. 2020).

Stroke duration did not differ significantly among the respondents, unlike findings by Odetunde et al. (2018, 2020). This may be attributed to the convenient sampling technique that was used, as all consenting people with stroke were included irrespective of the stroke duration. There was an equal distribution of gender (16 males and 16 females) among respondents like that of Odetunde et al.’s (2020) study that had an equal gender distribution of seven males and seven females. This may again be because convenience sampling was used where all available participants at the facility were recruited.

Ischaemic stroke was the most common (78.1%) followed by haemorrhagic (21.9%). Although the pre-validation sample was small, the findings support other studies that demonstrated a higher percentage of people suffer from ischaemic stroke (Donkor 2018; Mbui et al. 2018; Puthenpurakal & Crussell 2017). Further, 63% of the participants presented with body weakness on the left side, while 37% had right-side weakness. This finding contrasts with other studies that reveal the left hemispheric stroke as the most common as compared to right hemispheric stroke (Hedna et al. 2013; Odetunde et al. 2018). The inconsistency may be because of the small sample of participants or peculiarity of the population in the geographic location of the health facility where data were collected.

The Swahili version of SSQOL scale demonstrated the same evidence of content and equivalence to the original SSQOL scale. This is confirmed through the findings of domain mean scores of the English and Swahili versions of the SSQOL scales, which did not differ except for the vision domain. The vision domain reported slightly higher scores in the English SSQOL scale as compared to the Swahili version hence the variability. This may have been attributed to the contextual inappropriateness that was established during the adaptation process for item number 1 as well as the ambiguity of item number 2 of the vision domain. This finding partially concurs with that of Pedersen et al.’s (2018) study that demonstrated similar findings of ambiguity of item number 2 of the vision domain.

Further, respondents’ mean scores were lowest in the work, self-care, social roles, mobility, upper extremity and energy domains. This again agreed partially with the Hausa translation study that showed lower scores on the work domain (Odetunde et al. 2018). This was associated with loss of function, which is a major stroke sequela (Ganvir et al. 2018). The total mean score and s.d. of participants in the English and Swahili version of SSQOL were 82, s.d. = 31.04 and 81, s.d. = 20.24, respectively, which indicates a good understanding of questions in the Swahili version (Beaton et al. 2000).

### Strengths and limitations

The fact that most of the 42 ethnic communities of Kenya speak Swahili made the translation and adaptation process of SSQOL questionnaire into Swahili easy as there was ready and adequate information on the language that underpinned the achievement of the desired concept. However, there are limited HRQOL tools devised or culturally adapted and valid in Kenya that could be used to correlate with the responses to the Swahili questionnaire. Our study, therefore, had to rely on the original English of the SSQOL scale for comparability, which has not been contextualised in a Kenyan setting. In addition, studies of cross-cultural adaptation are new knowledge in Kenya; therefore, it was challenging to include sector-specific translators who are well-versed in translation.

### Recommendations

Further research should be conducted to investigate the reliability and validity of this SSQOL scale in Swahili.

## Conclusion

The original English SSQOL scale was translated and adapted to Swahili. The FSV of the SSQOL scale demonstrated the same constructs as the original English SSQOL scale. All items in the 12 domains were understood and captured well with the semantic and experiential equivalence fitting that of the Kenyan culture thus achieving content and face validity of the Swahili version.

## Appendix 1: The stroke-specific quality of life Swahili questionnaire.

### Hojaji Mahsusi ya Ubora wa maisha ya kiharusi

#### Sehemu A: Maelezo yanayokuhusu

**Weka tiki au jaza ndani ya nafasi zilizotolewa**

#### Sehemu ya B

**Sehemu hii inajumuisha vipengele 12 ambavyo vinapaswa kukamilishwa na mshiriki. Tafadhali soma na uelewe utaratibu wa kutuza alama kabla ya kujaza hojaji hii.**

**Alama: Kila ki pengele kitatuzwa alama kwa kuzingatia utaratibu ufuatao:**

**Table:**

Usaidizi wa kila kitu (Siwezi kitu chochote)	Ninakubali kabisa	1
Usaidizi mwingi (Matatizo mengi)	Ninakubali kwa kiasi	2
Baadhi ya usaidizi (Matatizo kiasi)	Sikubali wala kukataa	3
Usaidizi mdogo tu (Matatizo kidogo)	Ninakataa kwa kiasi	4
Hakuna usaidizi unaohitajika (Hakuna matatizo yoyote)	Ninakataa kabisa	5

#### Nguvu

**Table:**

1.	Nilihisi kuwa mchovu wakati mwingi	-----
2.	Nililazimika kuacha shuguli zangu ili kupumzika	-----
3.	Nilikuwa nimechoka kiasi cha kutofanya kazi nilizotaka kufanya	-----

#### Majukumu ya kifamilia

**Table:**

1.	Sikujiunga na familia yangu kujiburudisha.	-----
2.	Nilihisi kuwa mzigo kwa familia yangu.	-----
3.	Hali yangu ya afia iliathiri maisha yangu ya kibinafsi.	-----

#### Lugha

**Table:**

1.	Je, ulikuwa na matatizo kuzungumza? Kwa mfano, kukwama, kusitasita, kugugumiza au kukokoteza maneno yako?	-----
2.	Je, ulikuwa na matatizo kuzungumza na simu?	-----
3.	Je, watu wengine walikuwa na matatizo kukuelewa ukiongea?	-----
4.	Je, ulikuwa na matatizo ya kupata neno uliotaka kutamka?	-----
5.	Je, ulilazimika kujirudia ili watu wakuelewe?	-----

#### Kusongesha mwili

**Table:**

1.	Je, ulikuwa na matatizo ya kutembea? (Ikiwa mgonjwa hawezi kutembea, enda kwa swali la 4 na sahihisha maswali 2-3 kama 1.)	-----
2.	Je, ulipoteza usawa wa mwili wakati wa kuinama au kufikia kitu?	-----
3.	Je, ulipata shida kupanda ngazi za nyumba?	-----
4.	Je, ulilazimika kupumzika ukitembea au ukitumia kiti cha magurudumu?	-----
5.	Je, ulikuwa na matatizo kusimama?	-----
6.	Je, ulikuwa na matatizo kuinuka kutoka kwenye kiti?	-----

#### Hali ya hisia

**Table:**

1.	Nilipoteza matumaini ya maisha yangu yajayo	-----
2.	Sikutaka kuhusiana na watu au kufanya chochote.	-----
3.	Nilijitenga na watu wengine.	-----
4.	Nilijiamini kiasi tu kwa maisha yangu.	-----
5.	Sikuwa na hamu ya chakula.	-----

#### Nafsi

**Table:**

1.	Nilikuwa mwenye hasira	-----
2.	Nilikosa uvumilivu kwa wenzangu.	-----
3.	Nafsi yangu imebadilika.	-----

#### Kujitunza

**Table:**

1.	Je, ulihitaji usaidizi kuandaa chakula?	-----
2.	Je, ulihitaji usaidizi kula? Kwa mfano, kuchukua chakula kutoka kwa sahaniau kuweka chakula kwa mdomo?	-----
3.	Je, ulihitaji usaidizi kuvaa soksi au viatu, kufunga vifungu au kufunga zipu?	-----
4.	Je, ulihitaji usaidizi kuoga?	-----
5.	Je, ulihitaji usaidizi kutumia choo?	-----

#### Majukumu ya Kijamii

**Table:**

1.	Sikuenda kutembea mara nyingi kama ambavyo ningetaka.	-----
2.	Nilijiburudisha kwa muda mfupi zaidi ya ambavyo ningependa	-----
3.	Sikuwaona marafiki zangu wengi kama ambavyo ningependa.	-----
4.	Nilishiriki ngono mara chache tu kuliko ambavyo ningependa.	-----
5.	Hali yangu ya afia iliathiri maisha yangu ya kijamii.	-----

#### Fikra

**Table:**

1.	Ilikuwa vigumu sana kuwa makini.	-----
2.	Nilikuwa na matatizo kukumbuka mambo.	-----
3.	Ilinibidi niandike mambo ili kuyakumbuka.	-----

#### Matumizi ya mikono

**Table:**

1.	Je, ulikuwa na matatizo kuandika au kupiga chapa?	-----
2.	Je, ulikuwa na matatizo kuvaa soksi?	-----
3.	Je, ulikuwa na matatizo kufunga vifungu vya nguo?	-----
4.	Je, ulikuwa na matatizo kufunga zipu?	-----
5.	Je, ulikuwa na matatizo kufungua chupa?	-----

#### Kuona

**Table:**

1.	Je, ulikuwa na matatizo ya kutazama televisheni vizuri?	-----
2.	Je, ulikuwa na matatizo ya kufikia vitu kwa kukosa kouna?	-----
3.	Je, ulikuwa na matatizo ya kuona vitu kwa upande moja tu?	-----

#### Kazi

**Table:**

1.	Je, ulikuwa na matatizo kutekeleza kazi zako za kila siku nyumbani?	-----
2.	Je, ulikuwa na matatizo kukamilisha kazi ulizoanza?	-----
3.	Je, ulikuwa na matatizo kufanya kazi uliyokuwa unafanya hapo awali?	-----

JUMLA YA ALAMA

## References

[CIT0001] Beaton, D.E., Bombardier, C., Guillemin, F. & Ferraz, M.B., 2000, ‘Guidelines for the process of cross-cultural adaptation of self-report measures’, *Spine* 25(24), 3186–3191. 10.1097/00007632-200012150-00014 11124735

[CIT0002] Beaton, D., Bombardier, C., Guillemin, F. & Ferraz, M.B., 2002, ‘Recommendations for the cross-cultural adaptation of health status measures,’ *New York: American Academy of Orthopaedic surgeons* 12, 1–29.

[CIT0003] Bowden, A. & Fox-Rushby, J.A., 2003, ‘A systematic and critical review of the process of translation and adaptation of generic health-related quality of life measures in Africa, Asia, Eastern Europe, the Middle East, South America’, *Social Science & Medicine* 57(7), 1289–1306. 10.1016/S0277-9536(02)00503-8 12899911

[CIT0004] Chandran, P., Shenoy, D., Thavody, J. & Lilabi, M., 2017, ‘Assessment of quality of life of stroke survivors in a rural area of North Kerala, India’, *International Journal of Community Medicine and Public Health* 4(3), 841. 10.18203/2394-6040.ijcmph20170769

[CIT0005] Donkor, E.S., 2018, ‘Stroke in the 21st century: A snapshot of the burden, epidemiology, and quality of life’, *Stroke Research and Treatment* 2018(3), 1–10. 10.1155/2018/3238165 PMC6288566 30598741

[CIT0006] Ganvir, S., Harishchandre, M. & Kunde, C., 2018, ‘Validation of Marathi version of stroke-specific quality of life’, *Annals of Indian Academy of Neurology* 21(4), 290–293. 10.4103/aian.AIAN-449-17 30532359 PMC6238549

[CIT0007] Gbiri, C.A. & Akinpelu, A.O., 2013, ‘Relationship between post-stroke functional recovery and quality of life among Nigerian stroke survivors’, *The Nigerian postgraduate Medical Journal* 20(1), 29–33. 23661207

[CIT0008] Goerman, P.L. & Caspar, R.A., 2010, ‘A preferred approach for the cognitive testing of translated materials: Testing the source version as a basis for comparison’, *International Journal of Social Research Methodology* 13(4), 303–316. 10.1080/13645570903251516

[CIT0009] Gorelick, P.B., 2019, ‘The global burden of stroke: Persistent and disabling’, *The Lancet Neurology* 18(5), 417–418. 10.1016/S1474-4422(19)30030-4 30871943

[CIT0010] Guillemin, F., Bombardier, C. & Beaton, D., 1993, ‘Cross-cultural adaptation of health-related quality of life measures: Literature review and proposed guidelines’, *Journal of Clinical Epidemiology* 46(12), 1417–1432. 10.1016/0895-4356(93)90142-N 8263569

[CIT0011] Hall, D.A., Zaragoza Domingo, S., Hamdache, L.Z., Manchaiah, V., Thammaiah, S., Evans, C. et al., 2018, ‘A good practice guide for translating and adapting hearing-related questionnaires for different languages and cultures’, *International Journal of Audiology* 57(3), 161–175. 10.1080/14992027.2017.1393565 29161914

[CIT0012] Hawkins, M., Cheng, C., Elsworth, G.R. & Osborne, R.H., 2020, ‘Translation method is validity evidence for construct equivalence: Analysis of secondary data routinely collected during translations of the Health Literacy Questionnaire (HLQ)’, *BMC Medical Research Methodology* 20(1), 1–13. 10.1186/s12874-020-00962-8 PMC7249296 32456680

[CIT0013] Hawkins, M., Elsworth, G.R. & Osborne, R.H., 2018, ‘Application of validity theory and methodology to patient-reported outcome measures (PROMs): Building an argument for validity’, *Quality of Life Research* 27(7), 1695–1710. 10.1007/s11136-018-1815-6 29464456 PMC5997725

[CIT0014] Hedna, V.S., Albany, N., Bodhit, A.N., Ansari, S. & Waters, M., 2013, ‘Hemispheric differences in ischemic stroke : Is left-hemisphere stroke more common ?’, *Journal of Clinical Neurology* 9(2), 97–102 10.3988/jcn.2013.9.2.97 23626647 PMC3633197

[CIT0015] Kibui, A.W., 2014, ‘Language policy in Kenya and the New Constitution for Vision 2030,’ *International Journal of Educational Scien ce and Research* 4(5), 89–98.

[CIT0016] Kyte, D.G., Calvert, M., Van der Wees, P.J., Ten Hove, R., Tolan, S. & Hill, J.C., 2015, ‘An introduction to patient-reported outcome measures (PROMs) in physiotherapy’, *Physiotherapy (United Kingdom)* 101(2), 119–125. 10.1016/j.physio.2014.11.003 25620440

[CIT0017] Lisanza, V., 2021, ‘Swahili gaining popularity globally’, in *Un*, viewed 07 July 2022, from https://www.un.org/africarenewal/magazine/december-2021/swahili-gaining-popularity-globally.

[CIT0018] Mbui, J., Kaduka, L., Korir, A., Kwasa, J., Wabwire, S., Kisiangani, I. et al., 2018, ‘Stroke distribution patterns and characteristics in Kenya’s leading public health tertiary institutions: Kenyatta National Hospital and Moi Teaching and Referral Hospital’, *Cardiovascular Journal of Africa* 29(2), 68–72. 29745965 10.5830/CVJA-2017-046 PMC6008906

[CIT0019] Michira, J.N. & Iribemwangi, P.I., 2014, ‘Kiswahili as an official language in Kenya: Its past, Present and Future roles and challenges,’ In *Reyono Journal of Interdisciplinary Studies* 3.

[CIT0020] Moshki, M., Khajavi, A., Vakilian, F., Minaee, S. & Hashemizadeh, H., 2019, ‘The content comparison of health-related quality of life measures in heart failure based on the international classification of functioning, disability, and health: A systematic review’, *Journal of Cardiovascular and Thoracic Research* 11(3), 167–175. 10.15171/jcvtr.2019.29 31579455 PMC6759616

[CIT0021] Muralidharan, P.C., Kumar, V., Chitra, G., Sreejith, K. & Gafoor, S.A., 2019, ‘Quality of life in post stroke patients with early rehabilitation intervention: A cross sectional study’, *Journal of Medical Science and Clinical Research* 7(8), 66–75.

[CIT0022] Odetunde, M.O., Akinpelu, A.O. & Odole, A.C., 2017, ‘Validity and reliability of a Nigerian-Yoruba version of the stroke-specific quality of life scale 2.0’, *Health and Quality of Life Outcomes* 15(1), 1–12. 10.1186/s12955-017-0775-9 29052510 PMC5649048

[CIT0023] Odetunde, M.O., Akinpelu, A.O. & Odole, A.C., 2018, ‘Cross-cultural adaptatiion and validation of the stroke specific quality of life 2.0 scale into hausa language’, *Journal of Patient-Reported Outcomes* 2(1), 1–14. 10.1186/s41687-018-0082-1 30574661 PMC6301903

[CIT0024] Odetunde, M.O., Odole, A.C., Odunaiya, N.A., Odetunde, N.A., Okoye, E.C., Mbada, C.E. et al., 2020, ‘Cross-cultural adaptation and validation of the IGBO language version of the stroke-specific quality of life scale 2.0’, *Pan African Medical Journal* 37(111), 1–14. 10.11604/pamj.2020.37.111.19557 PMC7755362 33425144

[CIT0025] Pedersen, S.G., Heiberg, G.A., Nielsen, J.F., Friborg, O., Stabel, H.H., Anke, A. et al., 2018, ‘Validity, reliability and Norwegian adaptation of the Stroke-Specific Quality of Life (SS-QOL) scale’, *SAGE Open Medicine* 6, 205031211775203. 10.1177/2050312117752031 PMC5764138 29344360

[CIT0026] Puthenpurakal, A. & Crussell, J., 2017, ‘Stroke 1: Definition, burden, risk factors and diagnosis’, *Nursing Times* 113(11), 43–47.

[CIT0027] Sallam, S.A., Al-Khamis, F.A., Muaidi, Q.I. & Abdulla, F.A., 2019, ‘Translation and validation of the stroke specific quality of life scale into Arabic’, *NeuroRehabilitation* 44(2), 283–293. 10.3233/NRE-182552 31006693

[CIT0028] Shakya, D., Chaudhary, R., Shakya, D. & Shakya, B., 2019, ‘Quality of life and disability in stroke survivors’, *Journal of Karnali Academy of Health Sciences* 2(3), 227–233.

[CIT0029] Tawa, N., Brink, Y., Rhoda, A., Urimubenshi, G., Giljam-Enright, M., Charumbira, M.Y. et al., 2021, *Collaborative capacity development to complement stroke rehabilitation in Africa* (Vol. 1), viewed n.d., from https://books.aosis.co.za/index.php/ob/catalog/book/85

[CIT0030] Wagner, A.K., Wyss, K., Gandek, B., Kilima, P.M., Lorenz, S. & Whiting, D., 1999, ‘A Kiswahili version of the SF-36 Health Survey for use in Tanzania: Translation and tests of scaling assumptions’, *Quality of Life Research* 8(1–2), 101–110. 10.1023/A:1026441415079 10457743

[CIT0031] Wayessa, D.I., Chala, M.B., Demissie, S.F., Adebe, A.B., Janakiraman, B., Deme, S. et al., 2022, ‘Cross-cultural translation, adaptation, and validation of the stroke-specific quality of life scale into Amharic language,’ *Research square*. Preprint. 10.21203/rs.3.rs-1681816/V1 PMC9869570 36691045

[CIT0032] Williams, L.S., Weinberger, M., Harris, L.E., Clark, D.O. & Biller, J., 1999, ‘Development of a stroke-specific quality of life scale’, *Stroke* 30(7), 1362–1369. 10.1161/01.STR.30.7.1362 10390308

[CIT0033] Winstein, C.J., Stein, J., Arena, R., Bates, B., Cherney, L.R., Cramer, S.C. et al. 2016, ‘Guidelines for adult stroke rehabilitation and recovery: A guideline for healthcare professionals from the American Heart Association/American Stroke Association’, *Stroke* 47(6), e98–e169. 10.1161/STR.0000000000000098 27145936

[CIT0034] Yamane, T., 1967, Elementary Sampling Theory, Prentice-Hall, Viewed n.d., from https://books.google.co.za/books/about/Elementary_sampling_theory.html?id=RuFLAAAAMAAJ&redir_esc=y.

[CIT0035] Yangatimbi, E., Zobanga, J.K., Grégbia, S.S., Kinima, J. & Mbelesso, P., 2020, ‘Epidemiological aspects of stroke in young people at the Friendship University Hospital Center in Bangui in the Central African Republic’, *Neuroscience and Medicine* 11(4), 91–99. 10.4236/nm.2020.114011

