# Corrigendum: Translation and adaptation of the stroke-specific quality of life scale into Swahili

**DOI:** 10.4102/sajp.v79i1.1934

**Published:** 2023-07-10

**Authors:** Emily M. Nyanumba, Joseph M. Matheri, Nassib Tawa, Patrick M. Mburugu

**Affiliations:** 1 Department of Physiotherapy, Faculty of Rehabilitative Sciences, Jomo Kenyatta University of Agriculture and Technology, Nairobi, Kenya; 2 Department of Physiotherapy, Faculty of Rehabilitation, Kenya Medical Training College, Nairobi, Kenya; 3 Centre for Research in Spinal Health and Rehabilitation Medicine, Department of Rehabilitation Sciences, College of Health Sciences, Jomo Kenyatta University of Agriculture and Technology, Nairobi, Kenya; 4 Department of Child Health and Paediatrics, School of Medicine, Jomo Kenyatta University of Agriculture and Technology, Nairobi, Kenya

In the published article, Nyanumba, E.M., Matheri, J.M., Tawa, N. & Mburugu, P.M., 2023, ‘Translation and adaptation of the stroke-specific quality of life scale into Swahili’, *South African Journal of Physiotherapy* 79(1), a1847. https://doi.org/10.4102/sajp.v79i1.1847, there was an error regarding the affiliation for Emily M. Nyanumba. As well as having affiliation Department of Physiotherapy, Faculty of Rehabilitation, Kenya Medical Training College, Nairobi, Kenya, they should also have Department of Physiotherapy, Faculty of Rehabilitative Sciences, Jomo Kenyatta University of Agriculture and Technology, Nairobi, Kenya.

The authors apologise for this error. The correction does not change the study’s findings of significance or overall interpretation of the study’s results or the scientific conclusions of the article in any way.

